# A Rapid and Efficient Building Block Approach for Click Cyclization of Peptoids

**DOI:** 10.3389/fchem.2020.00405

**Published:** 2020-05-19

**Authors:** Mamidi Samarasimhareddy, Mai Shamir, Deborah E. Shalev, Mattan Hurevich, Assaf Friedler

**Affiliations:** ^1^Institute of Chemistry, The Hebrew University of Jerusalem, Jerusalem, Israel; ^2^Wolfson Centre for Applied Structural Biology, The Hebrew University of Jerusalem, Jerusalem, Israel; ^3^Department of Pharmaceutical Engineering, Azrieli College of Engineering Jerusalem, Jerusalem, Israel

**Keywords:** peptides, peptide cyclization, peptoids, peptide-peptoid hybrids, click chemistry, peptide backbone modification

## Abstract

Cyclic peptide-peptoid hybrids possess improved stability and selectivity over linear peptides and are thus better drug candidates. However, their synthesis is far from trivial and is usually difficult to automate. Here we describe a new rapid and efficient approach for the synthesis of click-based cyclic peptide-peptoid hybrids. Our methodology is based on a combination between easily synthesized building blocks, automated microwave assisted solid phase synthesis and bioorthogonal click cyclization. We proved the concept of this method using the INS peptide, which we have previously shown to activate the HIV-1 integrase enzyme. This strategy enabled the rapid synthesis and biophysical evaluation of a library of cyclic peptide-peptoid hybrids derived from HIV-1 integrase in high yield and purity. The new cyclic hybrids showed improved biological activity and were significantly more stable than the original linear INS peptide.

## Introduction

Linear peptides have several disadvantages as drug leads, such as metabolic instability due to enzymatic degradation, hydrolysis or oxidation, short half-life and rapid clearance, poor oral bioavailability and in some cases poor solubility and low membrane permeability (Marqus et al., 2017). Peptide cyclization is a useful strategy for overcoming these disadvantages. Cyclization is an approach borrowed from nature that is used to restrict the peptide conformation and by doing so improve the peptide affinity and selectivity to the target proteins (Craik et al., 2013; Fosgerau and Hoffmann, 2015; Zorzi et al., 2017). Cyclic peptides can be classified into head-to-tail, head-to-side chain, side chain-to-tail, and side chain-to-side chain cyclization (Zhang et al., 2019). Several approaches have been used to generate cyclic peptides, such as backbone cyclization (Gilon et al., 1991), peptide stapling (Schafmeister et al., 2000), and native chemical ligation (Dawson et al., 1994). These structures can be formed with chemically stable bonds, such as an amide, lactone, ether, thioether, or disulfide bonds (Qvit et al., 2017). Peptide cyclization can be achieved by using native amino acids side chains, including cysteine disulfide bridges (Góngora-Benítez et al., 2014), amine arylation and alkylation (Lautrette et al., 2016), etc. (Tang et al., 2017; Zhang et al., 2019). In addition, reactions including non-native amino acids are also commonly used, such as ring-closing metathesis (RCM) (Lecourt et al., 2018), click chemistry and more. The restricted conformation of cyclic peptides is more protected against degradation and chemical modifications and thus the stability of cyclic peptides is enhanced (Barreto et al., 2016; Dougherty et al., 2019). The number of cyclic peptides entering clinical trials or already approved is drastically increasing (White and Yudin, 2011; Jing and Jin, 2020). For example, cyclosporine A has been used for the treatment of allografts due to its immunosuppressive activity since its approval by the Food and Drug Administration (FDA) in 2001 (Matsuda and Koyasu, 2000; Shin et al., 2016; Gang et al., 2018). Romidepsin was approved by the FDA for cutaneous T-cell lymphoma in 2009 and for peripheral T-cell lymphomas in 2011 (Zorzi et al., 2017; Gang et al., 2018).

Another approach for improving the pharmacological properties of peptides is converting peptides into peptidomimetics by introducing peptide bond mimics (Moore and Matsoukas, 1995; Qvit et al., 2017; Mabonga and Kappo, 2020). For example, incorporating D-amino acids or unnatural amino acids into biologically active peptides can improve metabolic stability. The unnatural amino acid sarcosine was used in the development of Saralasin, an angiotensin II analog, which is used as a partial agonist of the angiotensin II receptor, treating hypertension. Substitution to the unnatural amino acid sarcosine supported resistance to aminopeptidase degradation, which improved the bioactivity of the compound (Disease et al., 2001; Qvit et al., 2017). Peptoids are a class of peptidomimetics consisting of N-substituted glycine oligomers (Simon et al., 1992; Lau, 2014). Unlike in peptides, the side chains in peptoids are connected to the backbone nitrogen and not to the chiral α-carbon (Simon et al., 1992; Dohm et al., 2011). Peptoids are highly stable toward proteolysis and are able to fold into well-defined secondary structures (Norgren et al., 2009; Culf and Ouellette, 2010; Dohm et al., 2011; Zabrodski et al., 2015).

There is an urgent need for developing new and rapid synthetic routes for combining peptide cyclization and peptide bond mimetics. Combining peptide cyclization with peptoids can result in significant improvement of the pharmacological properties of a lead peptide (Fowler et al., 2008; Silva et al., 2015; Barreto et al., 2016; Furukawa et al., 2016; Lam et al., 2017). For example, incorporation of peptoid residues into cyclic peptides improves the cell permeability compared to all peptidic or all peptoidic sequences. Several reports presented the synthesis of cyclic peptide-peptoid hybrids (Ostergaard and Holm, 1997; Shin et al., 2007; Avan et al., 2014; Lee and Lim, 2014; Kaniraj and Maayan, 2015). For example, a series of cyclic peptide-peptoid scaffolds that included diverse peptoid side chains were tested for passive membrane permeability using parallel artificial membranes. The variants were inherently permeable irrespective of the functional groups on their side chains (Furukawa et al., 2016). An example of highly selective cyclic peptide-peptoid hybrids was shown for inhibitors of the injectisome T3SS without affecting the flagellar T3SS (Lam et al., 2017). Cyclic peptide-peptoid hybrids were also implemented as bacterial quorum sensing modulators (Fowler et al., 2008), melanocortin receptor agonists and histone deacetylase inhibitors (Ovadia et al., 2010; Murugan et al., 2013). These molecules have many advantages over simple peptides or peptoids in terms of structural diversity and biological function. Thus, developing new and rapid synthetic methods for the synthesis of cyclic peptide-peptoid hybrids can also be used for the high throughput preparation of libraries.

Copper (I)-promoted [3+2] Huisgen cycloaddition of azides with alkynes (CuAAC) is one of the most efficient click reactions with widespread applications in organic chemistry and drug discovery (Bock et al., 2007; Best, 2009; Gehringer and Laufer, 2017). Click chemistry is an efficient tool for linking peptides with radioactive molecules or fluorescent compounds, other bioactive molecules, such as small molecules that serve as drugs or nucleic acids, and also for peptide cyclization. The CuAAC reaction is widely used because this reaction is very robust, selective, insensitive to changes in pH and temperature and can be performed in the presence of natural functional groups (Ahmad Fuaad et al., 2013; Testa et al., 2014). Microwave assisted (MW) synthesis can enhance and expedite the formation of the triazole using the typical click reaction protocols. MW assisted click was used in preparation of peptide and carbohydrate conjugates using the click reaction (Wang et al., 2003; Zabrodski et al., 2015).

**Figure 1:**
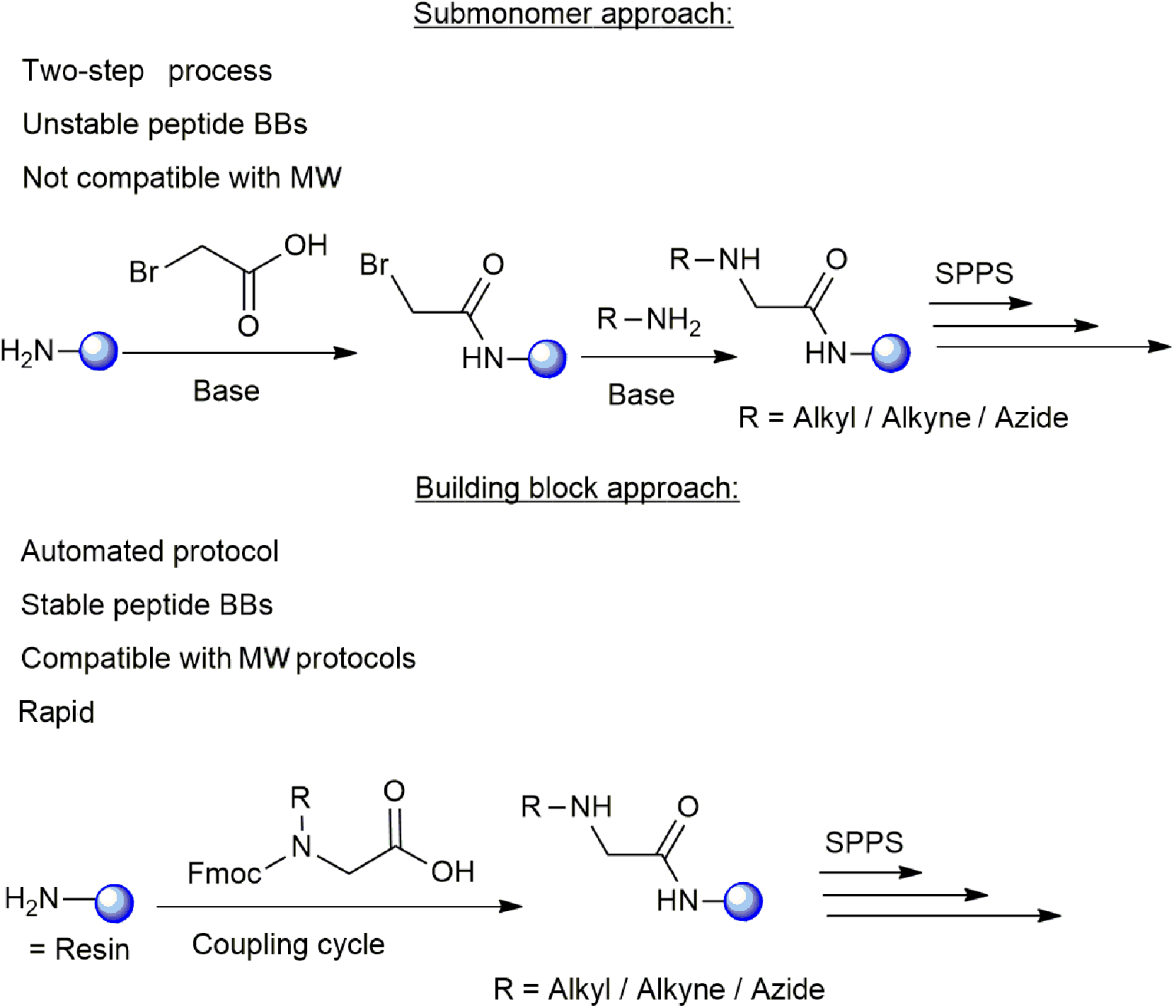
Illustration of the submonomer and building block approaches used for the synthesis of cyclic peptide-peptoid hybrids. The main advantages and limitations of each approach are presented. The resin is shown as a blue sphere.

The synthesis of cyclic peptide-peptoid hybrids using click chemistry can be carried out by two different approaches: the submonomer approach and the building block approach. The submonomer approach was reported in several cases and includes a two-step procedure: (i) coupling of bromoacetic acid to the free amine at the N-terminus of the resin-bound growing protected peptide chain, (ii) alkylation with the desired amine (Figure 1; Zuckermann et al., 1992; Zuckermann and Kodadek, 2009; Sarma and Kodadek, 2012; Park et al., 2013). Hundreds of primary amines are currently available commercially and can be used for synthesizing peptide-peptoid libraries by the submonomer approach. However, the existing submonomer approach protocols require several synthetic steps in which reagents are added manually for each alkylation, thus the strategy cannot be fully automated (Olivos et al., 2002; Richter et al., 2007). In addition, the alkyl amines with low boiling points with short carbon chains are not compatible for MW-assisted synthesis protocols and some of them are unavailable commercially. On the other hand, the building block approach is performed by coupling the protected N-alkylated amino acids to the peptide sequence in one step, like any other standard protected amino acid. This approach enables the incorporation of peptoid building blocks (BBs) into the standard solid phase peptide synthesis (SPPS) protocols in an automated manner with the advantages of the use of MW. The major difficulty in such a synthesis is the preparation of diverse BBs with suitable protecting groups, which also should be compatible with MW-SPPS conditions.

To address the above challenges, we describe the development of a new and rapid synthetic approach, which may provide the desired cyclic peptide-peptoid hybrids within several hours in an almost completely automated manner. To achieve this, we synthesized azide and alkyne N-alkylated amino acids, incorporated them into the peptide sequence using SPPS, and cyclized the peptide using click chemistry protocols that were optimized for MW-SPPS. The peptide selected for proving the concept of the new cyclization method is the HIV-1 integrase (IN) derived peptide IN_181−188_. This is a short derivative of the IN-activating peptide developed in our lab and termed INS (derived from IN residues 174-188), which we have shown to kill HIV-infected cells by inducing multi-integration and apoptosis (Levin et al., 2010). Alanine scan revealed that the truncated peptide IN_181−188_ binds and activates IN protein as efficiently as INS itself and thus it was used instead of the long parent peptide (Gabizon et al., 2013).

## Results

### Synthesis of Peptoid Building Blocks

**Figure 2:**
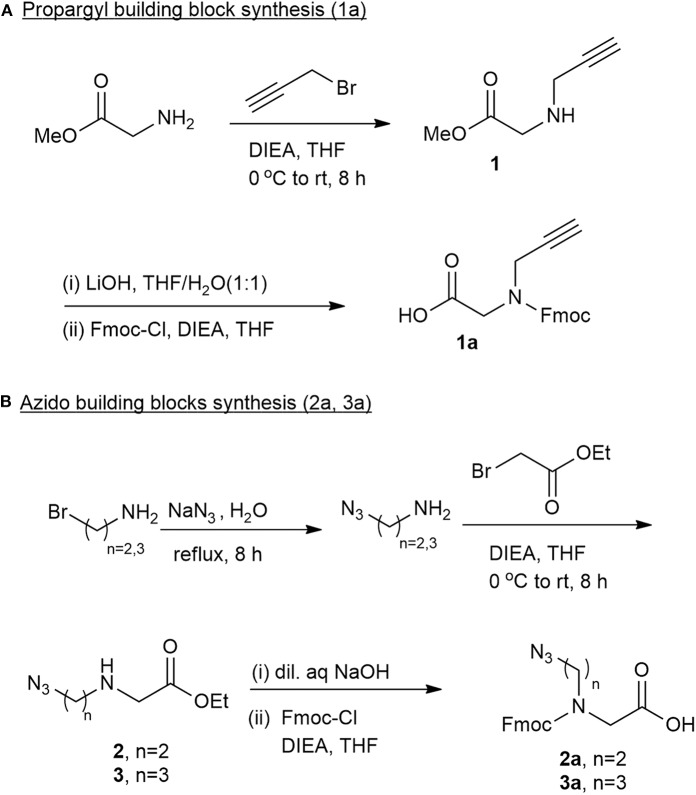
Fmoc protected peptoid building blocks synthesized and used in this study. **(A)** Synthesis of propargyl building block **1a**. **(B)** Synthesis of azido building blocks. **2a** contains two carbons in the backbone and **3a** contains three carbons in the backbone.

To synthesize a diverse library of cyclic peptide-peptoids hybrids, there was a need for the corresponding peptoid building blocks that would be incorporated into the peptide sequence. Three unnatural Fmoc-protected peptoid BBs with different chain lengths were designed and prepared. These BBs can be inserted readily into a desired location in a peptide sequence, similar to regular amino acids, employing standard automated microwave Fmoc-SPPS procedures. Both the azido and alkyne peptoid BBs were prepared by developing a new synthetic strategy (Figure 2). The synthesis of the propargyl BB **1a** was performed by reacting glycine methyl ester with propargyl bromide in the presence of diisopropylethylamine (DIEA) and dry tetrahydrofuran (THF). Several other bases such as triethylamine and pyridine were also tested, but DIEA gave the best yield of the crude intermediate **1**. The purity of the intermediate, measured by thin layer chromatography (TLC), was 80–85%. Hydrolysis using lithium hydroxide and Fmoc protection of the amine in the presence of DIEA provided the desired Fmoc protected alkyne building block **1a** (Figure 2A). The crude N-propargyl glycine **1a** was further triturated with ether and recrystallized from dichloromethane (DCM) and hexane to obtain the pure compound.

The two N-azidoalkyl glycine building blocks were synthesized using similar protocols. Sodium azide treatment of 2-bromo-ethylamine and 3-bromopropyl amine under reflux provided the 2-azidoethylamine and 3-azideopropyl amine as colorless oils that were dissolved in THF, cooled and treated with 1.0 equivalents of ethylbromoacetate to provide the stable ester intermediates 2**-**azido ethyl-N-glycine methyl ester **2** and the 3-azidopropyl-N-glycine methyl ester **3**, respectively. A simultaneous reaction of hydrolysis in 4 M NaOH and Fmoc protection (after adjusting the pH to 8.0–8.5) provided the Fmoc-azido ethyl-N-glycine **2a** and Fmoc-azidopropyl-N-glycine **3a**, respectively (Figure 2B). Ether trituration followed by recrystallization in DCM and hexane provided pure compounds **2a** and **3a** ready to be used for SPPS.

### Synthesis of Peptide-Peptoid Hybrids Derived From IN_181−188_

**Figure 3:**
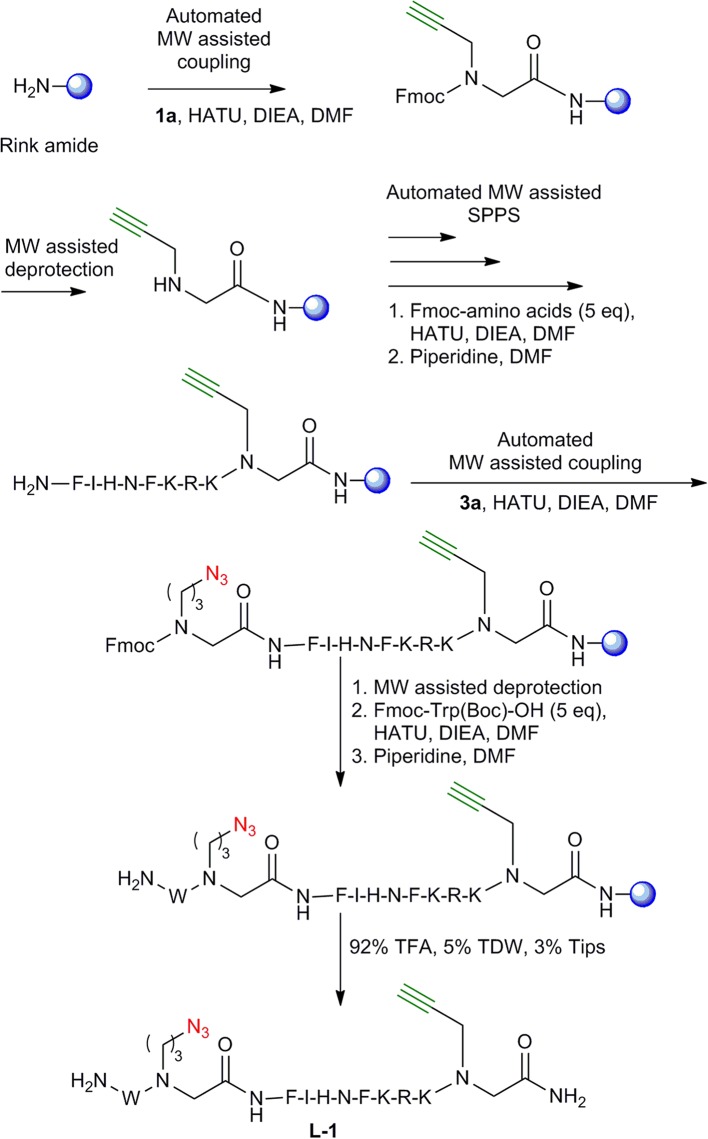
Synthesis of **L-1**. Synthesis of **L-1** peptide-peptoid hybrid employing MW- assisted SPPS. The resin is shown as a blue sphere.

**Figure 4:**
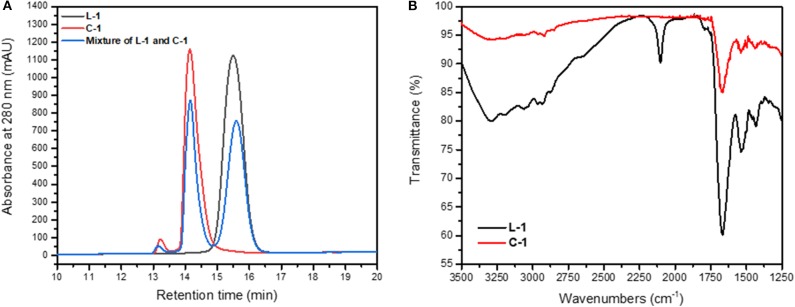
Cyclization of the **L-1** peptide. **(A)** Overlay of RP-HPLC of **L-1** (black), **C-1** (red) and a mixture of both (blue). The **L-1** and **C-1** peptides eluted at different times from C18 column. **(B)** IR analysis of **L-1** peptide showing the presence of azide peak at 2,104 cm^−1^, which is absent in the IR spectrum of the **C-1**.

To test the compatibility of the new building blocks with SPPS, we first synthesized linear peptide-peptoid hybrids derived from IN_181−188_. As a model, the MW-assisted automated synthesis of **L-1** was performed using building blocks **1a** and **3a** (Figure 3). The linear peptide **L-2** was prepared following a similar strategy only that **1a** and **3a** were introduced in a reverse order to that described for **L-1**. **1a** was introduced at the C-terminus and **3a** was coupled between the phenylalanine and the tryptophan at the N-terminal. **L-3** was prepared using **1a** and **2a**. The alkyne functionalized building block **1a** was coupled at the C-terminus and the azide functionalized BB **2a** on the N-terminus. For the synthesis of **L-4, 1a** and **2a** were also introduced but with the azide building block on the C-terminus and the alkyne on the N-terminus. After coupling the tryptophan, the Fmoc was removed and the linear peptides were cleaved and analyzed by Reverse Phase-High Performance Liquid Chromatography (RP-HPLC) (Figure 4), Electrospray Ionization-Mass Spectrometry (ESI-MS) and Infra-red spectroscopy (IR).

### Developing a Cyclization Protocol for the Peptide-Peptoid Hybrids

**Figure 5:**
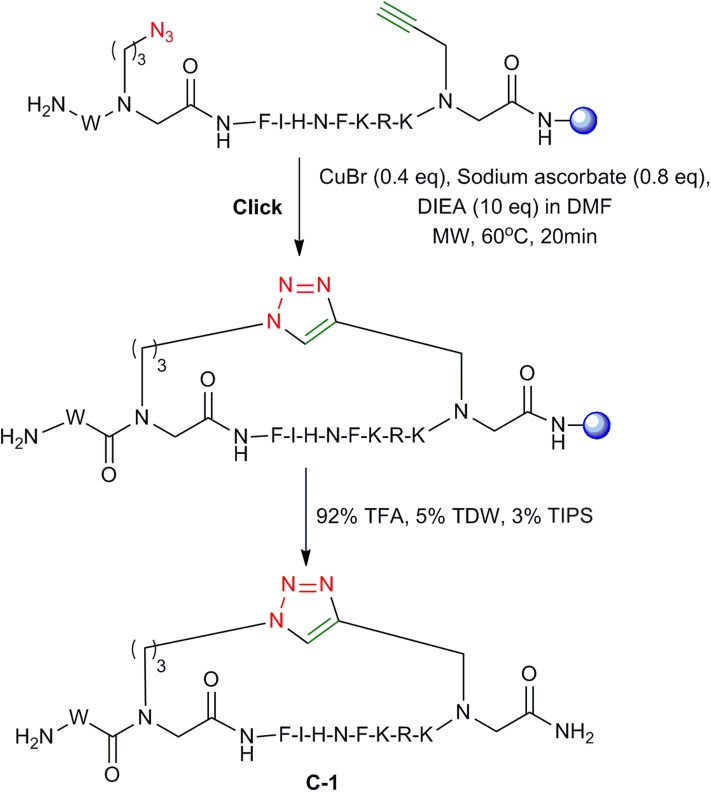
On-resin click cyclization. On-resin click cyclization of peptide-peptoid hybrids employing MW conditions. The resin is shown as a blue sphere.

To optimize the protocol for the cyclization of the peptide-peptoid hybrids, the linear peptide **L-1** was synthesized again as described above and different conditions for on-resin MW assisted cyclization were tested. A solution of CuBr, sodium ascorbate, and DIEA dissolved in DMF was added to the resin with the linear peptide **L-1** and cyclization was performed at different temperatures, reaction times and using different solvent systems. At 80°C, the cyclization was completed in approximately 15 min as monitored by RP-HPLC. However, several unidentified impurities were observed and the overall yield was unsatisfactory. At 40°C, the reaction took more than 1 h. The optimal conditions that resulted in the highest yield were using MW irradiation at 60°C for 20 min with the power of 25 W (Figure 5). RP-HPLC indicated that cyclization using these conditions resulted in >95% reactant **L-1** to product **C-1** conversion.

The formation of the cyclic peptide-peptoid hybrids was monitored by RP-HPLC (Figure 4A). All the cyclic peptides eluted from the RP-HPLC column with lower retention times than their linear analogs and were characterized by IR (Figure 4B) and ESI-MS. IR analysis showed a peak at 2,000–2,200 cm^−1^, which corresponds to the azido of **L-1**. This peak is not detectible in the IR spectrum of the **C-1** peptide, indicating successful cyclization (Figure 4B).

**Figure 6:**
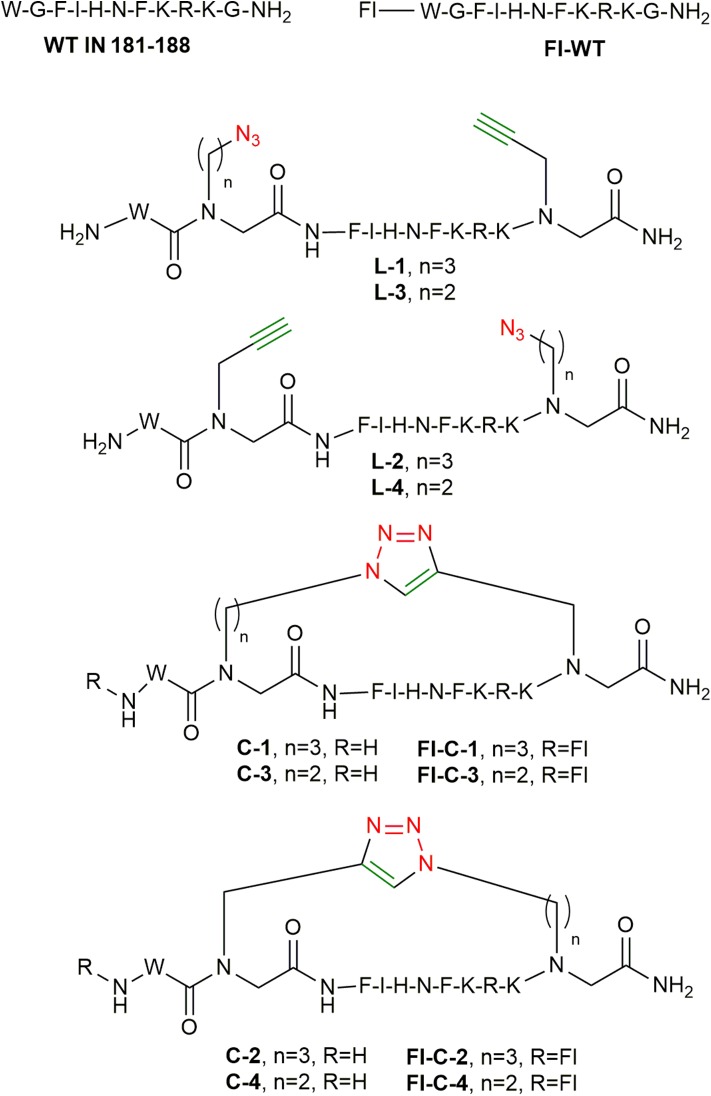
Peptides synthesized in this study.

After the optimization of the cyclization conditions for the model cyclic peptide **C-1**, the three other cyclic peptides, **C-2**, **C-3**, and **C-4**, were synthesized from their linear parent peptides on solid support prior to cleavage. The structures and sequences of the final cyclic products were predetermined based on the position at which the azide and alkyne BBs, **1a**, **2a**, or **3a**, were inserted into the peptide (Figure 6).

### Synthesis of Fluorescently Labeled Cyclic Peptides

**Figure 7:**
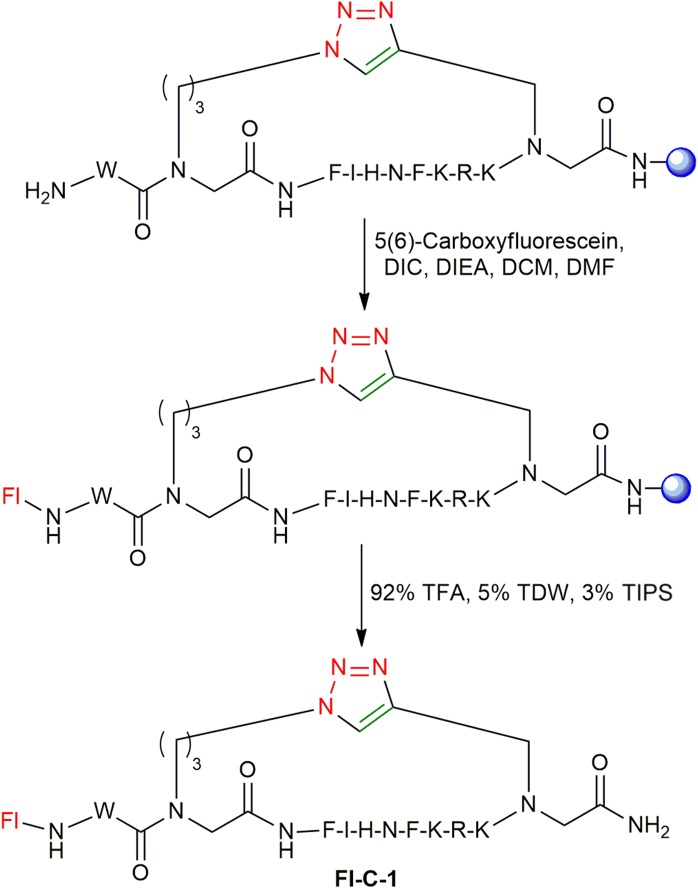
Fluorescein labeling of **C-1**. The resin is shown as a blue sphere.

After optimizing the automated strategy for synthesizing the linear peptides **L-1** to **L-4** and the cyclization to get **C-1** to **C-4**, fluorescein was coupled to the N-terminus of the cyclic peptides on the solid support as described (Figure 7; Weber et al., 1998). The fluorescein-labeled cyclic peptides **Fl-C-1–4** were cleaved and purified by HPLC and characterized by MS.

### Binding of the IN_181−188_ Cyclic Peptide-Peptoid Hybrids to IN

**Figure 8:**
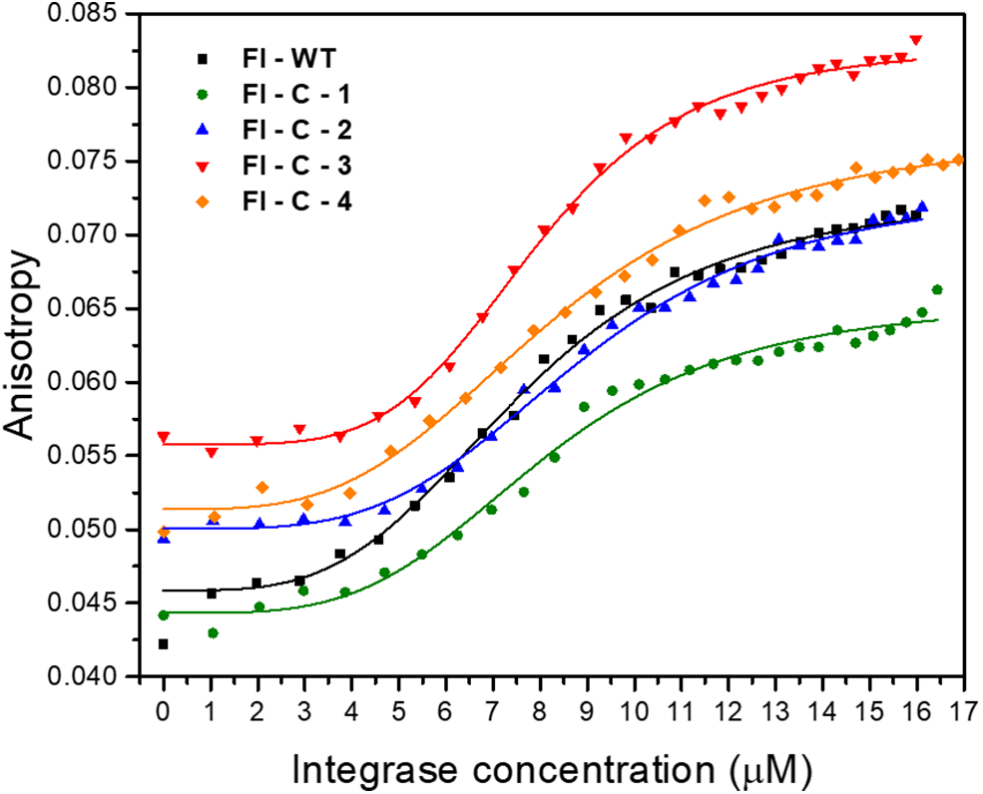
Fluorescence anisotropy binding studies of IN_181−188_ peptides to IN. Binding curves and fitting of linear Fl-WT (black), and Fl-C-1 (green), Fl-C-2 (blue), Fl-C-3 (red), and Fl-C-4 (orange) to IN are shown.

To test the activity of the cyclic peptide-peptoid hybrids, we first expressed and purified IN_1−288_ F185K/C250S as previously described (Gabizon et al., 2013). The double mutant protein was used since it has similar activity as the wild-type but higher solubility (Jenkins et al., 1996). Binding of the fluorescein-labeled cyclic peptides **Fl-C-1–4** to IN was evaluated using fluorescence anisotropy and compared to the binding to fluorescein labeled linear IN_181−188_ peptide (**WT**), Fl-WT IN_181−188_. The Hill coefficient for all the cyclic peptides was approximately 4, indicating cooperative binding of the peptides to tetrameric IN. The K_d_ values for IN binding to all four cyclic peptides were similar to the K_d_ of IN binding to the Fl-WT IN_181−188_, indicating that the cyclic peptides retained their affinity to the target protein, despite the more restricted conformation relative to the **WT** and the entropic penalty caused by cyclization (Figure 8, Table 1).

**Table 1:** Measured dissociation constants and Hill coefficient for binding of the IN_181−188_ cyclic peptides to IN.

**Peptide**	**K_**d**_ [μM]**	**Hill coefficient (*n*)**
Fl-WT IN_181−188_	7.6 ± 0.2	3.6 ± 0.4
Fl-C-1	8.1 ± 0.2	3.8 ± 0.4
Fl-C-2	9.0 ± 0.2	3.8 ± 0.3
Fl-C-3	7.7 ± 0.5	2.8 ± 0.5
Fl-C-4	7.0 ± 0.2	2.5 ± 0.2

### The Effect of the Peptides on IN Oligomerization

**Figure 9:**
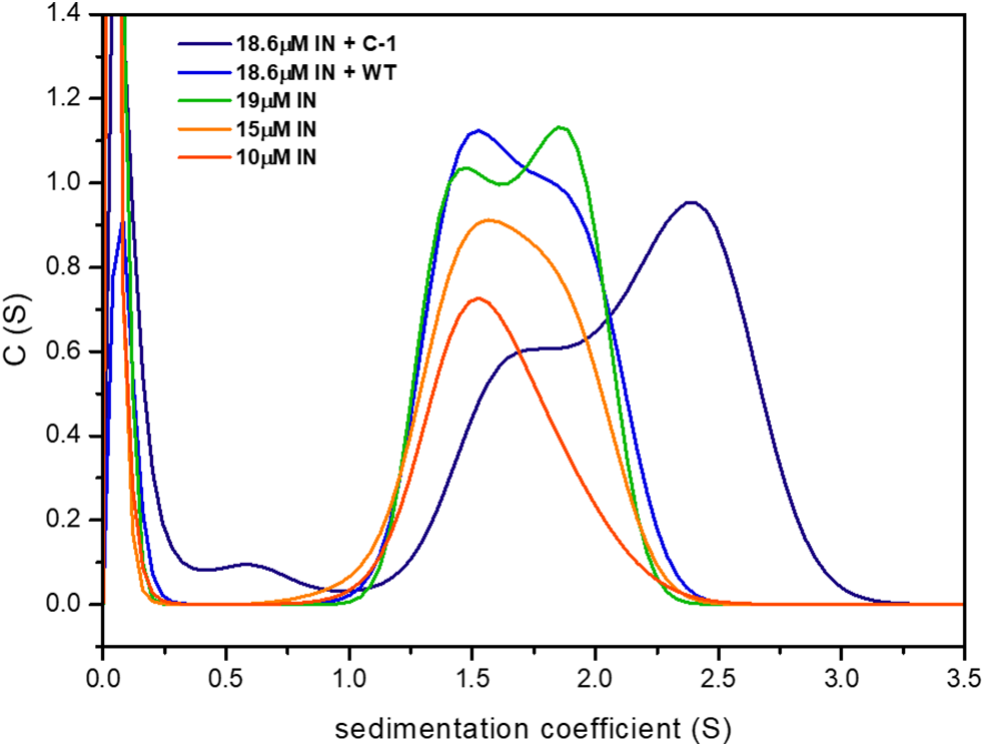
**C-1** shifts IN to larger oligomeric states compared to the WT peptide. Shown are the sedimentation curves for IN at three different concentrations, with and without the presence of **WT** and **C-1**. IN formed high order oligomers with higher sedimentation coefficients.

To examine the effect of IN_181−188_ peptides on IN oligomerization, we performed AUC sedimentation velocity experiments of IN at different concentrations in the presence and absence of the linear **WT** peptide and the cyclic **C-1** peptide. Higher IN concentrations resulted in a peak at larger S values, indicating the formation of a larger oligomer. The **WT** peptide stabilized a smaller oligomer that corresponds to an S value of approximately 1.5, while the cyclic peptide **C-1** shifted the sedimentation coefficient of IN from between 1.5 and 2 to a higher coefficient of 2.5, indicating the formation of a higher-order oligomer/aggregate (Figure 9).

### The Cyclic Peptide-Peptoid Hybrids Modulate IN Activity

**Figure 10:**
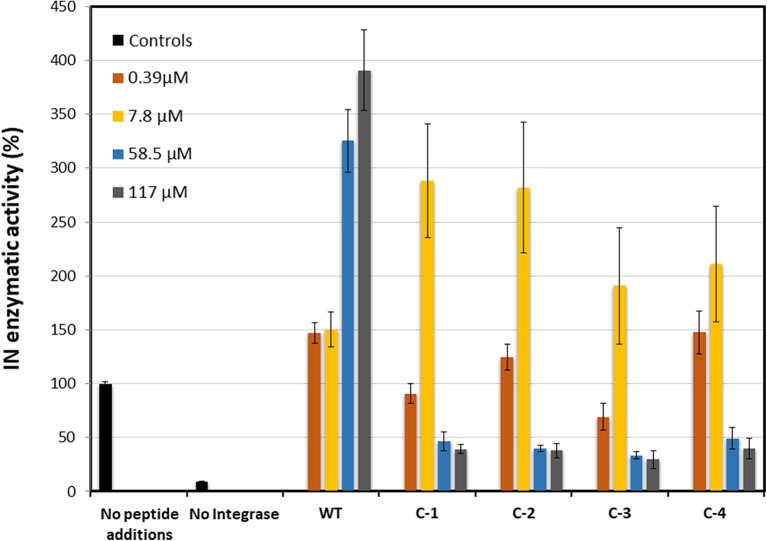
IN enzymatic activity in the presence of IN_181−188_ peptides. Four concentrations 0.39 μM (orange), 7.8 μM (yellow), 58.5 μM (blue), and 117 μM (gray) were measured for each peptide. The positive control (without peptides) and negative control (without IN) were measured as blank (black). All cyclic IN_181−188_ peptides were compared to the linear WT IN_181−188_.

The activity of the IN_181−188_ peptides was tested using HIV-1 Integrase commercial assay kit (XpressBio). The cyclic IN_181−188_ peptides **C-1** to **C-4** and **WT** were tested at four different concentrations of 0.39, 7.8, 58.5, and 117 μM. The **WT** peptide showed dose-dependent activation of IN in correlation with the peptide concentration, as previously reported by us (Levin et al., 2010). At the low concentrations of 0.39 and 7.8 μM the IN activity was increased by 50% and at the higher concentrations of 58.5 and 117 μM the activity increased by more than 200%. On the other hand, the **C-1** to **C-4** peptides showed around 150% increased activation at 7.8 μM but caused IN aggregation at 58.8 and 117 μM, resulting in significant loss of activity (Figure 10).

### Stability of the IN_181−188_ Cyclic Peptide-Peptoid Hybrids

**Figure 11:**
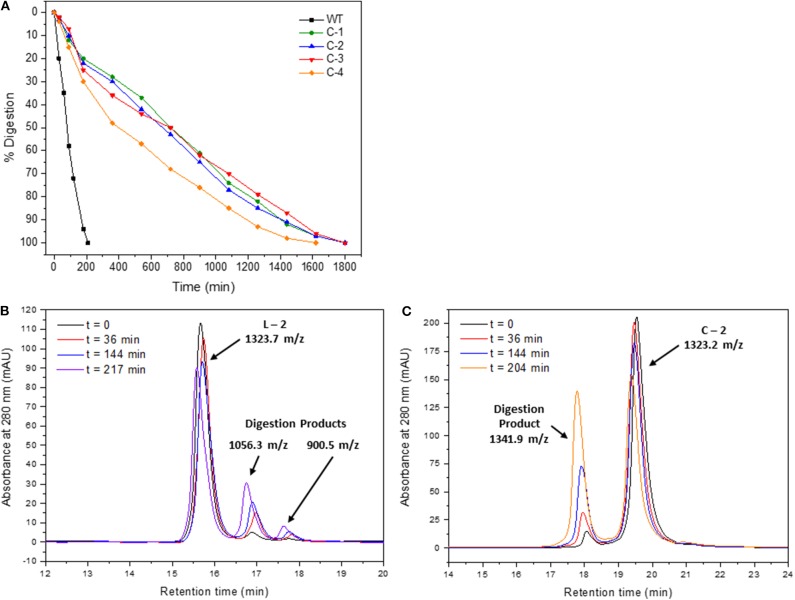
Stability measurements of cyclic IN_181−188_ peptides. **(A)** Trypsin digestion of **WT** (black) and **C-1** to **C-4** (green, blue, red, and yellow) with time. Trypsin digestion of **L-2 (B)** and **C-2 (C)** recorded by RP-HPLC at time intervals from *t* = 0 (black) for several hours (red, blue, purple, orange). The measured m/z of each peak is presented.

The stability of all four cyclic IN_181−188_ peptides **C-1**-**C-4** was tested using trypsin digestion and was compared to the **WT** and a linear precyclic precursor (Figure 11). The digestion rate was monitored by analytical RP-HPLC and showed that while **WT** was completely digested in around 2.5 h, all the cyclic peptides remained stable for about 30 h (Figure 11A). The digestion of the precyclic peptide **L-2** was compared to that of the cyclic analog **C-2**. **L-2** digestion showed mainly the digestion product G(*N*-propargyl)FIHNFKR but also the shorter fragment G(*N*-propargyl)FIHNFK, indicating the **L-2** peptide was hydrolyzed in two adjacent positions. The precyclic **L-2** peptide was more stable than the **WT** peptide toward proteolytic activity, because it contains N-alkylated amino acids (Figure 11B). The digestion of the cyclic **C-2** peptide led to the formation of only one product, which had an m/z value that was higher by 18 mass units compared to the cyclic peptide (Figure 11C). No fragments with lower m/z were observed in the digestion of **C-2**. The trypsin digestion studies provided strong evidence for the presence of a cyclic bridge in **C-2** because while the digestion of **L-2** led to the formation of smaller fragments corresponding to a truncated peptide, only one product with higher molecular weight was observed when the cyclic peptides were enzymatically cleaved. The mass increase of 18 Da indicates the addition of water as a result of the proteolytic process while the triazole bridge holds the entire peptide, preventing it from forming additional fragments.

## Discussion

In the current study, we developed a procedure that expedites the preparation of cyclic peptide-peptoid hybrids using a set of synthetic building blocks, automated MW-assisted SPPS and on-resin MW-assisted click chemistry protocols. Combining click chemistry and the peptide-peptoid cyclization with automated protocols is the key parameter that leads to the easy and efficient synthesis.

### A New Synthetic Method for Preparing Cyclic Peptide-Peptoid Hybrids

The strategy developed here is highly effective and provides the cyclic peptide-peptoid hybrids within 2–3 h with cyclization at the desired site of the peptide. The only extra step required here compared to standard automated MW-SPPS is the click reaction after the synthesis of the linear peptides with the azido and alkyne BBs at the desired locations. Adding the click solution to the resin and additional irradiation for 20 min at 60°C provides the cyclic peptide-peptoid hybrids **C-1–4**. This procedure not only decreases the number of steps but also accelerates the cyclic peptide synthesis itself. A library of cyclic analogs of a target peptide can be synthesized in just a few days.

The synthesis of the linear peptides **L-1**-**L-4** was performed using a fully automated approach employing standard MW-assisted SPPS. N-alkylated amino acids are known to be sterically hindered and usually it is not trivial to introduce them or couple the subsequent amino acids to them (Hurevich et al., 2007). Highly reactive reagents and elevated temperatures have been commonly used to overcome these difficulties. However, many of these reagents, such as the use of triphosgene as an activating reagent, are not compatible with an automated synthesis approach (Hurevich et al., 2007). This hampers attempts to automate the synthesis, making the production of peptide libraries that contain difficult coupling steps very tedious. MW-assisted SPPS, which utilizes standard protocols and can be automated, has been used to overcome difficult coupling steps that result from steric hindrance or from aggregations (Pratesi et al., 2017). However, in many of these cases, the MW procedure cannot be optimized and these steps are performed manually. Expediting the synthesis of the **L-1–L-4** peptide library was enabled by using the MW-assisted automated SPPS approach. Since the automated MW-assisted approach utilizes a high temperature as a standard practice, the same conditions were used to overcome the difficult coupling steps of and to the sterically hindered, N-alkylated building blocks **1a**, **2a**, and **3a**. The advantage of the strategy is that there is no need to perform any of the coupling steps manually and the entire process leading to the cyclic peptide is conducted automatically without interruption. In this process, only the cyclization and cleavage steps were performed manually. The same strategy was also used to expedite the synthesis of the **C-1–4** and **Fl-C-1–4** libraries.

Choosing to rely on a click reaction for the preparation of the library has many advantages. First, the alkyne and azide functionalized glycine BBs proved compatible with all standard SPPS protocols. Second, the standard protecting groups and the linker were fully immune to click reaction conditions so cyclization could be performed on the fully protected pre-cyclic peptide while still on the support. The on-resin cyclization strategy also prevents the problem of dimerization and oligomerization which is more prone to happen in solution phase click cyclization. Third, the triazole group and the entire cyclic peptide proved stable to standard cleavage conditions. The ability to prepare analogs with reverse and diverse architectures simply by changing the order in which the building blocks are introduced into the peptide sequence has many advantages compared to other cyclization methods e.g., amide, urea, disulfide, etc. In other cyclization methods the manipulation of protecting groups prior to the assembly of the peptide and before the cyclization steps is a major disadvantage (Davies, 2003). Choosing click chemistry proved compatible with the entire process since no additional deprotection or cleavage steps have to be performed prior to the cyclization. The optimized protocol for MW-assisted cyclization is an additional improvement that helps making cyclic peptide - peptoid hybrids more accessible. It allowed us to synthesize a diverse set of cyclic peptides quickly and efficiently. Furthermore, it overcame the tedious effort of extensive protecting group manipulation that is required for cyclization using other methods.

### Stability of the Cyclic Peptides to Tryptic Digestion Offers an Advantage Over the WT

Stability of cyclic and N-alkylated peptide-peptoid hybrids toward enzymatic degradation is one of the most significant advantages that can be gained by using this strategy and is crucial for improving the pharmacological properties. Our tryptic digestion studies show that both the precyclic and the cyclic hybrids are significantly more stable toward enzymatic hydrolysis than the **WT** peptide. Several molecular and structural features of the cyclic peptide-peptoid hybrids combine to provide this higher stability. The cyclic peptides in the library have non-native side chains, two backbone amides that are alkylated, and they are cyclized using a bond that is not naturally recognized by enzymes. Each of these features can contribute separately to the stability but their combination can enhance stability even more. Our results show that the contribution of the N-alkylation itself, as demonstrated for **L-2**, provides significant improvement in stability compared to the **WT** and dramatically increases its half life time. The cyclization provides further advantage because the triazole bridge holds the peptide from disassembly to shorter fragments even after hydrolysis, since it is not recognized by the enzyme.

### The Activity of IN in the Presence of IN_181−188_ Derived Cyclic Peptides

The cyclic peptides bound IN with a similar affinity to the linear parent **WT** peptide. The catalytic activity of IN in the presence of **C-1** and **C-2** at 7.8 μM was stimulated to a larger extent when compared to the stimulation caused by the **WT** peptide. At high peptide concentrations all four cyclic peptides stimulated IN aggregation, as was confirmed by AUC results (Figure 9). The **WT** and the cyclic peptides bound IN with the same affinity. At low concentrations, the **WT** and cyclic peptides stimulate IN activity, as previously described for the linear INS peptide (Levin et al., 2010). **C-1** and **C-2** activated IN to a larger extent than **C-3** and **C-4**. At higher peptide concentrations, the **WT** stimulated IN activity while the cyclic peptides induced IN aggregation resulting in loss of activity. We conclude that using the cyclic peptides at low concentrations is optimal—combining affinity, activity, and stability while avoiding aggregation.

In summary, the strategy of using readily synthesized Fmoc-protected peptoid building blocks in combination with automated MW-assisted SPPS and MW-assisted on-resin orthogonal cyclization offers quick access to cyclic peptide-peptoid hybrids. This approach provided four cyclic peptide-peptoid hybrids in a very short time, and without additional steps compared to the present strategies. Our approach can be useful for peptide-based drug discovery studies which require a rapid method for synthesizing libraries of stable and potent drug candidates.

## Materials and Methods

### Peptide Synthesis and On-Resin Cyclization

All peptides were synthesized on a Liberty Blue microwave assisted peptide synthesizer (CEM) using standard Fmoc-SPPS chemistry on rink amide with DIC/Oxyma as coupling reagents. The coupling of azide-functionalized building block **2a, 3a** and the alkyne-functionalized building blocks **1a** was performed using a mixture of HATU and DIEA in dimethylformamide (DMF) under MW conditions at 75°C for 5 min. Fmoc removal was performed at 90°C for 3 min using 20% piperidine in DMF during the entire synthesis. Click cyclization solution was made by dissolving 0.4 eq of CuBr, 0.8 eq of sodium ascorbate, and 10 eq of DIEA in DMF and was added to the resin with the linear peptides **L-1-4**. Cyclization was performed with MW irradiation at 60°C for 20 min with the power of 25 W (Figure 5). Cleavage of both linear and cyclic peptides performed using mixture of 92% TFA, 5% TDW, and 3% TIPS. The cleaved peptides were purified on a Merck-Hitachi HPLC using a reverse-phase C18 preparative column with a gradient range between 10 and 50% of acetonitrile (ACN) in TDW. ESI mass spectroscopy and analytical HPLC were used to check the identity and verify the peptide purity. All peptides were lyophilized and stored at −20°C. Fluorescein was coupled as described (Weber et al., 1998). A tryptophan residue was added at the N-terminus of the peptides when required for concentration measurements using UV spectroscopy.

Rink amide resin was placed in a CEM liberty blue synthesizer and was coupled with the azide-functionalized building block **3a** using a mixture of HATU and DIEA in dimethylformamide (DMF) under MW conditions at 75°C for 5 min. Fmoc removal was performed at 90°C for 3 min using 20% piperidine in DMF during the entire synthesis. The next eight amino acids were introduced in an automated way using standard MW-assisted SPPS protocols. After the coupling of phenylalanine, the alkyne functionalized building block **1a** was coupled using the same HATU and DIEA activation mixture as the **3a** and under the same MW conditions. The terminal Fmoc tryptophan was later coupled to provide the **L-1** linear peptoid-peptide hybrid (Figure 3).

### Fluorescence Anisotropy Binding Measurements

IN was titrated into a solution of 100 nM fluorescein labeled (Fl) IN_181−188_ cyclic peptides and into linear IN_181−188_, which served as a control. Anisotropy measurements were performed generally as described (Friedler et al., 2002) at 10°C with ionic strength of 1 M (adjusted by NaCl) and the binding curves were fit to the Hill equation (Figure 8, Table 1).

### AUC Measurements of IN With WT and C-1

Sedimentation velocity measurements were performed using a Beckman-Coulter ProteomeLab™ XL-I analytical ultracentrifuge. The IN was diluted to 10, 15, and 19 μM with 20 mM HEPES buffer containing 0.1 mM EDTA, 10% v/v glycerol, 1 mM DTT and an ionic strength of 1 M (with NaCl). 1.5 mM **C-1** and 4.2 mM linear **WT** were run in the same buffer in both the protein and reference solutions. All runs were done at 10°C for approximately 8 h at 45,000 rpm. The data was analyzed using SEDPHAT software by NIH.

### Proteolytic Digestion of L-2 WT and C-1-C-4

**WT** and **C-1–C-4** and **L-2** peptides, all synthesized without terminal Trp, were dissolved in 360 μL of HEPES 20 mM pH = 7.5 up to 450 μM. Then 7 μL of 8 μM trypsin were added to the peptide solution and the mixture was incubated at 37 °C. Every 30 min, 25 μL of the reaction solution were taken out and the residual trypsin activity quenched with 5 μL 2% TFA in 20% aqueous ACN. Fifty microliter of 10% aqueous acetonitrile was added to make the total volume 80 μL. Then the sample was run on reverse phase HPLC using a 5–60% ACN:Water gradient at room temperature (Hayouka et al., 2010; Tal-Gan et al., 2010). HPLC/MS of **C-2** digestion: peak at Rt 19.7 min: ESI-MS calculated for C_62_H_95_N_22_O_11_ [**C-2**+H]^+^ 1323.7; found: 1323.2. peak at Rt 18.0 min: ESI-MS calculated for C_62_H_97_N_22_O_12_ [digested **C-2**+H]^+^ 1341.6; found: 1341.9. HPLC/MS of **L-2** digestion: peak at Rt 15.7 min: ESI-MS calculated for C_62_H_95_N_22_O_11_ [**L-2**+H]^+^ 1323.7; found: 1323.3; peak at Rt 16.9 min: ESI-MS calculated for: C_51_H_74_N_15_O_10_ [G(*N*-propargyl)FIHNFKR+H]^+^ 1056.6; found: 1056.3; peak at Rt 17.7 min: ESI-MS calculated for C_45_H_62_N_11_O_9_ [G(*N*-propargyl)FIHNFK+H]^+^ 900.5; found: 900.5.

## Data Availability Statement

All datasets generated for this study are included in the article/supplementary material.

## Author Contributions

MSa, MSh, DS, MH, and AF conceived and designed the research. MSa, MSh, and DS performed the experiments and analyzed the data. MH and AF supervised the research. All authors contributed to the writing of the manuscript.

## Conflict of Interest

The authors declare that the research was conducted in the absence of any commercial or financial relationships that could be construed as a potential conflict of interest.

## References

[B1] Ahmad Fuaad A. A. H. Azmi F. Skwarczynski M. Toth I. (2013). Peptide conjugation via CuAAC “click” chemistry. Molecules 18, 13148–13174. 10.3390/molecules181113148 24284482 PMC6270195

[B2] Avan I. Hall C. D. Katritzky A. R. (2014). Peptidomimetics via modifications of amino acids and peptide bonds. Chem. Soc. Rev. 43, 3575–3594. 10.1039/c3cs60384a 24626261

[B3] Barreto A. D. F. S. Dos Santos V. A. Andrade C. K. Z. (2016). Synthesis of acylhydrazino-peptomers, a new class of peptidomimetics, by consecutive Ugi and hydrazino-Ugi reactions. Beilstein J. Org. Chem. 12, 2865–2872. 10.3762/bjoc.12.285 28144359 PMC5238544

[B4] Best M. D. (2009). Click chemistry and bioorthogonal reactions: Unprecedented selectivity in the labeling of biological molecules. Biochemistry 48, 6571–6584. 10.1021/bi9007726 19485420

[B5] Bock V. D. Speijer D. Hiemstra H. Van Maarseveen J. H. (2007). 1,2,3-Triazoles as peptide bond isosteres: synthesis and biological evaluation of cyclotetrapeptide mimics. Org. Biomol. Chem. 5, 971–975. 10.1039/b616751a 17340013

[B6] Craik D. J. Fairlie D. P. Liras S. Price D. (2013). The future of peptide-based drugs. Chem. Biol. Drug Des. 81, 136–147. 10.1111/cbdd.12055 23253135

[B7] Culf A. S. Ouellette R. J. (2010). Solid-phase synthesis of N-substituted glycine oligomers (α-peptoids) and derivatives. Molecules 15, 5282–5335. 10.3390/molecules15085282 20714299 PMC6257730

[B8] Davies J. S. (2003). The cyclization of peptides and depsipeptides. J. Pept. Sci. 9, 471–501. 10.1002/psc.491 12952390

[B9] Dawson P. Muir T. Clark-Lewis I. Kent S. (1994). Synthesis of proteins by native chemical ligation. Science 266, 776–779. 10.1126/science.7973629 7973629

[B10] Disease I. H. Failure H. Gavras H. Brunner H. R. (2001). Role of angiotensin and its inhibition in hypertension. Hypertension 37, 342–345. 10.1161/01.HYP.37.2.342 11230297

[B11] Dohm M. T. Kapoor R. Barron A. E. (2011). Peptoids: bio-inspired polymers as potential pharmaceuticals. Curr. Pharm. Des. 17, 2732–2747. 10.2174/138161211797416066 21728985

[B12] Dougherty P. G. Sahni A. Pei D. (2019). Understanding cell penetration of cyclic peptides. Chem. Rev. 119, 10241–10287. 10.1021/acs.chemrev.9b00008 31083977 PMC6739158

[B13] Fosgerau K. Hoffmann T. (2015). Peptide therapeutics: current status and future directions. Drug Discov. Today 20, 122–128. 10.1016/j.drudis.2014.10.003 25450771

[B14] Fowler S. A. Stacy D. M. Blackwell H. E. (2008). Design and synthesis of macrocyclic peptomers as mimics of a quorum sensing signal from *Staphylococcus aureus*. Org. Lett. 10, 2329–2332. 10.1021/ol800908h 18476747

[B15] Friedler A. Hansson L. O. Veprintsev D. B. Freund S. M. V. Rippin T. M. Nikolova P. V.. (2002). A peptide that binds and stabilizes p53 core domain: chaperone strategy for rescue of oncogenic mutants. Proc. Natl. Acad. Sci. U.S.A. 99, 937–942. 10.1073/pnas.241629998 11782540 PMC117409

[B16] Furukawa A. Townsend C. E. Schwochert J. Pye C. R. Bednarek M. A. Scott Lokey R. (2016). Passive membrane permeability in cyclic peptomer scaffolds is robust to extensive variation in side chain functionality and backbone geometry. J. Med. Chem. 59, 9503–9512. 10.1021/acs.jmedchem.6b01246 27690434

[B17] Gabizon R. Faust O. Benyamini H. Nir S. Loyterb A. Friedler A. (2013). Structure–activity relationship studies using peptide arrays: the example of HIV-1 Rev–integrase interaction. Med. Chem. Commun. 4, 252–259. 10.1039/C2MD20225E

[B18] Gang D. Kim D. W. Park H. (2018). Cyclic peptides: promising scaffolds for biopharmaceuticals. Genes 9:557. 10.3390/genes9110557 30453533 PMC6267108

[B19] Gehringer M. Laufer S. A. (2017). Click chemistry: novel applications in cell biology and drug discovery. Angew. Chem. Int. Ed. 56, 15504–15505. 10.1002/anie.201710195 29068506

[B20] Gilon C. Halle D. Chorev M. Selincer Z. Byk G. (1991). Backbone cyclization: a new method for conferring conformational constraint on peptides. Biopolymers 6, 745–750. 10.1002/bip.360310619 1718473

[B21] Góngora-Benítez M. Tulla-Puche J. Albericio F. (2014). Multifaceted roles of disulfide bonds. Peptides as therapeutics. Chem. Rev. 114, 901–926. 10.1021/cr400031z 24446748

[B22] Hayouka Z. Hurevich M. Levin A. Benyamini H. Iosub A. Maes M.. (2010). Cyclic peptide inhibitors of HIV-1 integrase derived from the LEDGF/p75 protein. Bioorg. Med. Chem. 18, 8388–8395. 10.1016/j.bmc.2010.09.046 20974536

[B23] Hurevich M. Barda Y. Gilon C. (2007). Synthesis of novel urea bridged macrocyclic molecules using BTC. Heterocycles 73, 617–625. 10.3987/COM-07-S(U)40

[B24] Jenkins T. M. Engelman A. Ghirlando R. Craigie R. (1996). A soluble active mutant of HIV-1 integrase. J. Biol. Chem. 271, 7712–7718. 10.1074/jbc.271.13.7712 8631811

[B25] Jing X. Jin K. (2020). A gold mine for drug discovery: strategies to develop cyclic peptides into therapies. Med. Res. Rev. 40, 753–810. 10.1002/med.21639 31599007

[B26] Kaniraj P. J. Maayan G. (2015). A facile strategy for the construction of cyclic peptoids under microwave irradiation through a simple substitution reaction. Org. Lett. 17, 2110–2113. 10.1021/acs.orglett.5b00696 25868085

[B27] Lam H. Schwochert J. Lao Y. Lau T. Lloyd C. Luu J.. (2017). Synthetic cyclic peptomers as type III secretion system inhibitors. Antimicrob. Agents Chemother. 61:e00060-17. 10.1128/AAC.00060-17 28652236 PMC5571333

[B28] Lau K. H. A. (2014). Peptoids for biomaterials science. Biomater. Sci. 2, 627–633. 10.1039/C3BM60269A 32481842

[B29] Lautrette G. Touti F. Lee H. G. Dai P. Pentelute B. L. (2016). Nitrogen arylation for macrocyclization of unprotected peptides. J. Am. Chem. Soc. 138, 8340–8343. 10.1021/jacs.6b03757 27332147 PMC6150454

[B30] Lecourt C. Dhambri S. Allievi L. Sanogo Y. Zeghbib N. Ben Othman R.. (2018). Natural products and ring-closing metathesis: synthesis of sterically congested olefins. Nat. Prod. Rep. 35, 105–124. 10.1039/C7NP00048K 29345263

[B31] Lee K. J. Lim H. S. (2014). Facile method to sequence cyclic peptides/peptoids via one-pot ring-opening/cleavage reaction. Org. Lett. 16, 5710–5713. 10.1021/ol502788e 25310875

[B32] Levin A. Hayouka Z. Helfer M. Brack-Werner R. Friedler A. Loyter A. (2010). Stimulation of the HIV-1 integrase enzymatic activity and cDNA integration by a peptide derived from the integrase protein. Biopolymers 93, 740–751. 10.1002/bip.21469 20517955

[B33] Mabonga L. Kappo A. P. (2020). Peptidomimetics: a synthetic tool for inhibiting protein–protein interactions in cancer. Int. J. Pept. Res. Ther. 26, 225–241. 10.1007/s10989-019-09831-5

[B34] Marqus S. Pirogova E. Piva T. J. (2017). Evaluation of the use of therapeutic peptides for cancer treatment. J. Biomed. Sci. 24:21. 10.1186/s12929-017-0328-x 28320393 PMC5359827

[B35] Matsuda S. Koyasu S. (2000). Mechanisms of action of cyclosporine. Immunopharmacology 47, 119–125. 10.1016/S0162-3109(00)00192-2 10878286

[B36] Moore G. J. Matsoukas J. M. (1995). Designing peptide mimetics. Rev. Clin. Pharmacol. Pharmacokinet. Int. Ed. 9, 53–58.

[B37] Murugan R. N. Park J. E. Lim D. Ahn M. Cheong C. Kwon T.. (2013). Development of cyclic peptomer inhibitors targeting the polo-box domain of polo-like kinase 1. Bioorganic Med. Chem. 21, 2623–2634. 10.1016/j.bmc.2013.02.020 23498919 PMC7561269

[B38] Norgren A. S. Budke C. Majer Z. Heggemann C. Koop T. Sewald N. (2009). On-resin click-glycoconjugation of peptoids. Synthesis 2009, 488–494. 10.1055/s-0028-1083302

[B39] Olivos H. J. Alluri P. G. Reddy M. M. Salony D. Kodadek T. (2002). Microwave-assisted solid-phase synthesis of peptoids. Org. Lett. 4, 4057–4058. 10.1021/ol0267578 12423085

[B40] Ostergaard S. Holm A. (1997). Peptomers: a versatile approach for the preparation of diverse combinatorial peptidomimetic bead libraries. Mol. Divers. 3, 17–27. 10.1023/A:1009698507588 9527474

[B41] Ovadia O. Linde Y. Haskell-Luevano C. Dirain M. L. Sheynis T. Jelinek R. (2010) The effect of backbone cyclization on PK/PD properties of bioactive peptide-peptoid hybrids: the melanocortin agonist paradigm. Bioorganic Med. Chem. 18, 580–589. 10.1016/j.bmc.2009.12.010. 20056544

[B42] Park M. Wetzler M. Jardetzky T. S. Barron A. E. (2013). A readily applicable strategy to convert peptides to peptoid-based therapeutics. PLoS ONE. 8:e58874. 10.1371/journal.pone.0058874 23555603 PMC3605428

[B43] Pratesi A. Stazzoni S. Lumini M. Sabatino G. Carotenuto A. Brancaccio D. (2017). Synthesis of dicarba-cyclooctapeptide Somatostatin analogs by conventional and MW-assisted RCM: a study about the impact of the configuration at C α of selected amino acids. Chem. Eng. Process. Process Intensif. 122, 365–372. 10.1016/j.cep.2017.02.005

[B44] Qvit N. Rubin S. J. S. Urban T. J. Mochly-Rosen D. Gross E. R. (2017). Peptidomimetic therapeutics: scientific approaches and opportunities. Drug Discov. Today 22, 454–462. 10.1016/j.drudis.2016.11.003 27856346 PMC5759319

[B45] Richter L. S. Spellmeyer D. C. Martin E. J. Figliozzi G. M. Zuckermann R. N. (2007). Combinatorial Peptide and Nonpeptide Libraries: A Handbook. (Wiley).

[B46] Sarma B. K. Kodadek T. (2012). Submonomer synthesis of a hybrid peptoid-azapeptoid library. ACS Comb. Sci. 14, 558–564. 10.1021/co3000852 22958123 PMC3676481

[B47] Schafmeister C. E. Po J. Verdine G. L. (2000). An all-hydrocarbon cross-linking system for enhancing the helicity and metabolic stability of peptides. J. Am. Chem. Soc. 122, 5891–5892. 10.1021/ja000563a

[B48] Shin J. M. Gwak J. W. Kamarajan P. Fenno J. C. Rickard A. H. Kapila Y. L. (2016). Biomedical applications of nisin HHS public access. J. Appl. Microbiol. 120, 1449–1465. 10.1111/jam.13033 26678028 PMC4866897

[B49] Shin S. B. Y. Yoo B. Todaro L. J. Kirshenbaum K. (2007). Cyclic peptoids. J. Am. Chem. Soc. 129, 3218–3225. 10.1021/ja066960o 17323948

[B50] Silva E. H. B. Emery F. S. Del Ponte G. Donate P. M. (2015). Synthesis of some functionalized peptomers via Ugi four-component reaction. Synth. Commun. 45, 1761–1767. 10.1080/00397911.2015.1042591

[B51] Simon R. J. Kania R. S. Zuckermann R. N. Huebner V. D. Jewell D. A. Banville S.. (1992). Peptoids: a modular approach to drug discovery. Proc. Natl. Acad. Sci. U.S.A. 89, 9367–9371. 10.1073/pnas.89.20.9367 1409642 PMC50132

[B52] Tal-Gan Y. Freeman N. S. Klein S. Levitzki A. Gilon C. (2010). Synthesis and structure-activity relationship studies of peptidomimetic PKB/Akt inhibitors: the significance of backbone interactions. Bioorganic Med. Chem. 18, 2976–2985. 10.1016/j.bmc.2010.02.031 20347317

[B53] Tang J. He Y. Chen H. Sheng W. Wang H. (2017). Synthesis of bioactive and stabilized cyclic peptides by macrocyclization using C(sp3)-H activation. Chem. Sci. 8, 4565–4570. 10.1039/C6SC05530C 28936334 PMC5590095

[B54] Testa C. Scrima M. Grimaldi M. D'Ursi A. M. Dirain M. L. Lubin-Germain N. (2014). 1,4-disubstituted-[1,2,3]triazolyl-containing analogues of MT-II: design, synthesis, conformational analysis, and biological activity. J. Med. Chem. 57, 9424–9434. 10.1021/jm501027w 25347033 PMC4255721

[B55] Wang Q. Chan T. R. Hilgraf R. Fokin V. V. Barry Sharpless K. Finn M. G. (2003). Bioconjugation by copper(I)-catalyzed azide-alkyne [3 + 2] cycloaddition. J. Am. Chem. Soc. 125, 3192–3193. 10.1021/ja021381e 12630856

[B56] Weber P. J. A. Bader J. E. Folkers G. Beck-Sickinger A. G. (1998). A fast and inexpensive method for N-terminal fluorescein-labeling of peptides. Bioorganic Med. Chem. Lett. 8, 597–600. 10.1016/S0960-894X(98)00084-5 9871567

[B57] White C. J. Yudin A. K. (2011). Contemporary strategies for peptide macrocyclization. Nat. Chem. 3, 509–524. 10.1038/nchem.1062 21697871

[B58] Zabrodski T. Baskin M. Kaniraj P. J. Maayan G. (2015). Click to bind: microwave-assisted solid-phase synthesis of peptoids incorporating pyridine-triazole ligands and their copper(II) complexes. Synlett 26, 461–466. 10.1055/s-0034-1378938

[B59] Zhang Y. Zhang Q. Wong C. T. T. Li X. (2019). Chemoselective peptide cyclization and bicyclization directly on unprotected peptides. J. Am. Chem. Soc. 141, 12274–12279. 10.1021/jacs.9b03623 31314512

[B60] Zorzi A. Deyle K. Heinis C. (2017). Cyclic peptide therapeutics: past, present and future. Curr. Opin. Chem. Biol. 38, 24–29. 10.1016/j.cbpa.2017.02.006 28249193

[B61] Zuckermann R. N. Kerr J. M. Kent S. B. H. Moosf W. H. (1992). Efficient method for the preparation of peptoids [oligo(N-substituted glycines)] by submonomer solid-phase synthesis. J. Am. Chem. Soc. 114, 10646–10647. 10.1021/ja00052a076

[B62] Zuckermann R. N. Kodadek T. (2009). Peptoids as potential therapeutics. Curr. Opin. Mol. Ther. 11, 299–307. 19479663

